# The Role of Genomic Scores in the Management of Prostate Cancer Patients: A Comprehensive Narrative Review

**DOI:** 10.3390/cancers17142334

**Published:** 2025-07-14

**Authors:** Alessandro Viti, Leonardo Quarta, Paolo Zaurito, Alfonso Santangelo, Andrea Cosenza, Francesco Barletta, Simone Scuderi, Armando Stabile, Vito Cucchiara, Francesco Montorsi, Giorgio Gandaglia, Alberto Briganti

**Affiliations:** 1 Unit of Urology, Division of Oncology, Gianfranco Soldera Prostate Cancer Lab, Urological Research Institute (URI), IRCCS San Raffaele Scientific Institute, 20132 Milan, Italy; 2 Vita-Salute San Raffaele University, 20132 Milan, Italy

**Keywords:** prostate cancer, genomic classifier, polygenic risk score, genetic risk, prognosis

## Abstract

Genomic score testing is increasingly being integrated into the management of prostate cancer (PCa) to overcome the limitations of traditional clinical and pathological parameters. Genomic tools will represent essential components of precision medicine, supporting risk stratification, therapeutic decision-making, and personalized screening strategies. Genomic score tests can be broadly classified into two main categories: polygenic risk scores (PRSs) and tumor-derived genomic classifiers (GCs). While not yet standard in routine practice, several international guidelines recommend their selective use when results are likely to impact clinical management. PRSs estimate an individual’s susceptibility to PCa based on the cumulative effect of multiple low-penetrance germline genetic variants. These scores show promise in enhancing early detection strategies and identifying men at higher genetic risk who may benefit from tailored screening protocols. Tumor-based GCs assays provide prognostic information that complements conventional clinical and pathological parameters, and are used to guide treatment decisions, including eligibility for active surveillance (AS) or adjuvant therapy after treatment of the primary tumor. This review summarizes and analyzes the current evidence on genomic testing in PCa, with a focus on the available assays, their clinical applications, and their predictive and prognostic value across the disease spectrum. When integrated with clinical and pathological parameters, these tools have the potential to significantly enhance personalized care and should be increasingly considered in routine clinical practice.

## Simple Summary

Genomic score testing is increasingly used in prostate cancer management to improve risk assessment and guide treatment. Several studies assessed the prognostic role of tumor-derived genomic classifiers and polygenic risk scores. While not yet routine in clinical guidelines, both tools show promise for advancing precision medicine in prostate cancer by enhancing prognostic accuracy and personalizing care decisions.

## 1. Introduction

As the management of prostate cancer (PCa) continues to evolve toward a more personalized approach, genomic tools are playing an increasingly central role in the clinical decision-making process. Among these, two complementary categories have emerged: polygenic risk scores (PRSs), which estimate inherited susceptibility based on germline variation, and tumor-derived genomic classifiers (GCs), which inform prognosis and treatment decisions based on tumor biology [1,2,3,4,5,6,7]. PRSs estimate an individual’s PCa susceptibility and risk based on the cumulative effect of numerous low-penetrance common genetic variants from germline deoxyribonucleic acid (DNA). Genome-wide association studies (GWAS) have identified several susceptibility loci associated with increased risk of developing PCa, which might explain familiarity as a well-known risk factor [1,2,3]. Indeed, these loci are estimated to explain up to 39% of the familial risk, highlighting the substantial contribution of common genetic variants to disease heritability beyond high-penetrance genes such as *BRCA1/2*, *ATM*, *CHEK2*, *HOXB13*, and others [1,4]. Even though the use of these tools is not yet considered to be a standard in clinical guidelines and clinical practice, PRSs show promise in improving early detection strategies by identifying men at elevated genetic risk without monogenic hereditary syndromes who may benefit from tailored screening protocols. On the other hand, GCs (such as Decipher, Oncotype DX, and Prolaris) are based on gene expression profiling of PCa specimens obtained through biopsy or surgery. These tests have shown excellent independent prognostic information beyond conventional clinical and pathological parameters, and their use is increasing to guide therapeutic decisions. For instance, they can potentially help determine eligibility for active surveillance (AS) in low- or favorable intermediate-risk disease or indicate the potential benefit of adjuvant therapy following radical prostatectomy (RP) in the presence of high-risk features. Some of the current international guidelines recommend the selective use of these genomic score tools in cases where their results are expected to influence clinical management, though broader implementation is anticipated as evidence and accessibility increase [1,2,3]. This review aims to provide a practical and comprehensive overview of the currently available genomic tests for PCa and the key differences among them, to support test selection and patient counseling within routine clinical practice.

## 2. Review

Given the heterogeneity of methodologies, clinical endpoints, and validation designs across genomic tests, we opted for a narrative review to synthesize clinically relevant evidence. A narrative literature review was conducted using the Web of Science, Medline, PubMed, and Embase databases, covering all records from inception to May 2025. Additional references were identified through manual cross-referencing of the bibliographies of relevant articles. The search strategy included the following terms: “(Genomic Classifier OR GC) OR (Decipher OR PORTOS) OR (Prolaris OR CCP) OR (Oncotype DX GPS) OR (Polygenic Risk Score OR PRS) AND (prostate cancer OR PCa).” The most pertinent studies were selected to provide a comprehensive overview of the clinical role and utility of PRSs and tumor-derived GCs in patients with PCa.

## 3. Polygenic Risk Scores

### 3.1. From Monogenic Testing to PRSs

Genetic testing has traditionally been effective in monogenic diseases, where strong genotype–phenotype correlations enable clear clinical applications. With the advent of GWAS, hundreds of genetic loci have been associated with common complex diseases [5,6]. However, the small effect sizes of individual variants and the moderate heritability of many traits limit the predictive power of traditional genetic testing in this context [7]. To address these limitations, polygenic scores (PGSs) have emerged as promising tools to perform a more comprehensive genetic risk assessment and to estimate an individual’s genetic risk to complex traits. Also referred to as polygenic indexes (PGIs) or as PRSs when used in the context of disease, PRSs provide a single numerical value that reflects the cumulative effect of many genetic variants, most commonly single-nucleotide polymorphisms (SNPs). PRSs are constructed using effect sizes derived from GWAS, which estimate the association strength between each SNP and the phenotype of interest. The final score is calculated by summing the number of risk alleles across a set of SNPs, each weighted by its corresponding effect size [8]. Unlike monogenic variants, PRSs reflect the polygenic architecture of complex diseases and aim to improve risk stratification, particularly when combined with traditional clinical factors. The first landmark study introducing the PRS framework was published in 2009 by the International Schizophrenia Consortium, demonstrating that thousands of common alleles, each with a very small effect, contribute to schizophrenia risk [9]. Since then, PRSs have been developed for a wide range of diseases. To support their standardization, transparency, and accessibility, the PGS Catalog, developed by EMBL-EBI and the University of Cambridge, was launched in 2019 as an open-access database of published polygenic scores. The catalog currently includes over 4,850 PRSs, spanning a broad spectrum of traits and improving ancestral diversity in genomic data [10,11]. PRSs aim to predict disease incidence and can define marked increases in lifetime risk or earlier onset in individuals with high scores. Potential clinical applications include prognostic prediction, insights into etiology and disease subtypes; stratification of patients based on likely therapeutic benefit; and the identification of novel biomarkers and drug targets [12].

### 3.2. Clinical Applications and Validation of PRSs

Since the introduction of PRSs, numerous models have been developed and validated for PCa. Today, 87 PRSs with evaluation data (Stage E) are listed in the PGS Catalog, ranging from small models using only 4 SNPs [13] to genome-wide approaches based on over six million variants [14]. The first study to assess the clinical application of PRS in PCa was published in 2011, using data from the Stockholm-1 cohort. This work evaluated whether a 35-SNP score could help determine the need for prostate biopsy. Results showed that the genetic model reduced the number of unnecessary biopsies by 22.7%, while missing only 3% of aggressive cases, highlighting the potential utility of PRS in biopsy decision-making [15]. Subsequent research has expanded the number of risk variants included in PRS and validated these scores in increasingly large and diverse cohorts. In 2018, a GWAS involving over 140,000 men identified 63 new PCa risk loci, increasing the total number of susceptibility variants beyond 100. The resulting PRS showed strong stratification capacity, with men in the 90th to 99th percentile presenting a relative risk of 2.69 compared with the population average [2]. In 2021, a meta-analysis identified 269 independent risk variants and generated the widely adopted PRS269. Individuals in the top decile of PRS269 had a 5- to 7-fold increased risk of developing PCa compared to those in the lowest decile [3]. Later studies showed that PRS269 was significantly associated with cancer detection on biopsy, when added to the Prostate Biopsy Collaborative Group predictor, improved discrimination performance [16]. More recently, in the UK-based BARCODE1 study [17], a 130-SNP PRS was evaluated as a population screening tool. Among the 6300 men assessed, those in the top 10% were invited for MRI and biopsy, leading to a 40% cancer detection rate. Of note, PRSs are also being evaluated in the context of AS. The PRS-451 and PRS-400 were assessed in the Canary PASS cohort, which included 1220 patients undergoing AS. Higher PRS values were associated with an increased risk of disease upgrading and potentially with tumor multifocality, suggesting that PRS may support more refined risk stratification among candidates for AS [18]. As noted in international guidelines [19,20,21,22], PRSs are not currently recommended for routine clinical use. However, their adoption is expanding beyond the research context, with private companies such as Allelica [23] and Vitall [24] now offering PRS testing for several common diseases, reflecting growing interest in polygenic risk assessment as part of personalized health strategies.

### 3.3. Scientific Gap

A key scientific gap of current PRSs lies in the limited understanding of their performance across diverse ancestral groups. Most have been developed in European populations, raising concerns about applicability elsewhere. Zhu et al. [25] evaluated PRSs across 13 cancers in over 100,000 Chinese individuals, demonstrating the value of ancestry-specific models and the need for population-tailored approaches. Further studies are needed to assess performance in other ancestral and admixed populations. Moreover, it remains unclear what impact the widespread use of array-based PRSs may have on the detection and clinical interpretation of high-penetrance mutations, which are not characterized by these tools.

## 4. Tumor-Derived Genomic Classifiers

At present, three GC assays are commercially available and recommended by major clinical guidelines for use in localized PCa: Decipher, Oncotype DX Genomic Prostate Score (GPS), and Prolaris [19,20,21,22,26,27]. These tests differ in platform, gene targets, and clinical indication, but all, by assessing ribonucleic acid (RNA) expression profiles, aim to offer deeper molecular insight into tumor biology and to complement conventional risk factors, which include both clinical and pathological features (Table 1). In the pre-treatment setting, particularly for patients newly diagnosed on biopsy, all three tests support risk stratification by assessing tumor aggressiveness and guiding the choice between AS and definitive treatment [28,29,30,31,32,33]. Among them, Decipher provides additional prognostic value in favorable intermediate-risk cases [34], while Oncotype DX GPS uniquely reports on individual pathological components [33]. Following primary treatment, Prolaris and Decipher are the two classifiers applicable in the postoperative setting [21,22,23,24,25,26,27,28,29]. In particular, Decipher is the most extensively validated tool and is endorsed by clinical guidelines to inform decisions on adjuvant or salvage radiotherapy after RP [35,36,37,38,39].

In advanced and metastatic disease, Decipher is currently the only partially validated GC. It is evolving from a purely prognostic instrument to a tool with predictive utility, with emerging evidence suggesting it may help identify patients more likely to benefit from early systemic treatment intensification [36,40].

Their implementation in clinical practice could assist physicians in selecting which patients should intensify vs. de-intensify follow-up and treatments, in patient counseling and prognostication, thus facilitating the implementation of personalized medicine (Table 2).

### 4.1. Prolaris

Prolaris is an RNA-based gene expression assay developed by Myriad Genetics in 2011 to assess the risk of biochemical recurrence (BCR) and PCa-specific mortality (PCSM) following RP, radiotherapy (RT), or conservative management [30,41,42]. Prolaris is performed on formalin-fixed paraffin-embedded (FFPE) PCa tissue obtained from either diagnostic needle biopsy cores or RP specimens. The test quantifies the expression of 31 cell-cycle progression (CCP) genes, normalized against 15 housekeeping genes to account for technical variability. Gene expression is measured in triplicate using quantitative reverse transcription polymerase chain reaction (qRT-PCR), and the results are used to generate a Prolaris Molecular Score, also referred to as the CCP score [42]. Prolaris molecular score ranges from 1.8 to 8.7 [29,30,43].

The test can be applied in both the pre-treatment setting, to assist in clinical decision-making by identifying patients who may safely defer multimodality treatment and patients who can be managed with AS, and in the postoperative setting, where it helps determine the need for adjuvant treatment [29,43,44,45]. For example, according to the updated scoring framework, for patients classified as D’Amico low risk, a Prolaris score below 2.7 suggests less aggressive cancer than average, while a score above 3.7 indicates a more aggressive phenotype [29,46,47,48]. In routine clinical practice, the CCP score is often integrated with other clinical and pathological variables to enhance risk stratification. A common approach is its combination with the University of California, San Francisco Cancer of the Prostate Risk Assessment (CAPRA) score, resulting in the Clinical Cell-Cycle Risk (CCR) score. This composite index is calculated as: CCR = 0.39 × CAPRA + 0.57 × CCP [30]. The CCR score has been shown to significantly improve prognostic accuracy over the use of CAPRA or CCP alone. It is employed to estimate the 10-year risk of both metastatic progression and PCSM [31,43,44].

### 4.2. Oncotype DX GPS

The Oncotype DX GPS Assay, owned by MDxHealth, is a biopsy-based RT-PCR test developed in 2013 [49,50]. It was originally designed to assess the biological aggressiveness of localized PCa in the pre-treatment setting and support treatment decisions in men who are candidates for AS versus definitive therapy [33]. The assay analyzes RNA extracted from FFPE prostate needle biopsy specimens; unlike the other two GCs, it is exclusively performed on diagnostic biopsy tissue. It is based on the expression quantification of a 17-gene panel, including 12 cancer-related genes and 5 reference (housekeeping) genes. The cancer-associated genes reflect four critical biological pathways: androgen signaling, cellular organization, stromal response, and cellular proliferation, while the reference genes ensure internal control [50]. The test provides a GPS ranging from 0 to 100, where higher scores indicate more biologically aggressive tumor characteristics. The GPS has been clinically validated as a strong and independent predictor of adverse pathology, defined as Gleason score ≥ 4+3 and/or non-organ-confined disease, BCR, metastatic progression, and PCSM in patients with very low-, low-, and intermediate-risk disease [33,51,52]. GPS remains the only genomic test that provides specific risk estimates not only for overall adverse pathology but also for individual pathological components, such as pathologic stage ≥ pT3a and high-grade histology. In addition, the use of GPS has been associated with increased clinical recommendation and adoption of AS in men with very low-, low-, and favorable intermediate-risk PCa [32,53].

### 4.3. Decipher

Decipher Prostate GC is a 22-gene transcriptome-wide RNA microarray assay developed by GenomeDx (Biosciences, San Diego, United States [US]) in 2013 [36] and now owned by Veracyte, that assesses expression data across over 1.4 million RNA transcripts from both coding and non-coding genes. These 22 biomarkers include protein-coding and non-coding RNAs involved in cell proliferation, cell-cycle progression, immune response, cellular structure, adhesion, and motility, as well as three genes of unknown function [54]. The assay is performed on RNA extracted from FFPE PCa tissue, either from diagnostic needle biopsy cores or prostate resection specimens. Validation studies showed that high Decipher GC scores on RP specimens were predictors of metastasis and PCSM within five years following RP [36,38] and RT [55], demonstrating that an increase in GC score of 0.1 was associated with a significant rise in metastatic risk [37]. Later, the test was validated on diagnostic biopsy samples as a predictor of metastasis within ten years after RP and has also been shown to predict distant metastases following definitive radiation and androgen deprivation therapy (ADT) in patients with intermediate- and high-risk PCa [28,56]. The result of the Decipher test is expressed as a GC score, which ranges on a continuous scale from 0 to 1. This score is used to stratify patients into three distinct genomic risk categories: low risk (<0.45), intermediate risk (0.45–0.60), and high risk (>0.60), each associated with progressively increasing probabilities of disease progression and adverse outcomes. The Decipher prostate biopsy test report is designed to support clinical decision-making by providing individualized estimates for the patient’s 5- and 10-year risk of developing metastases when managed with standard-of-care treatment strategies. In addition, it reports the 15-year risk of PCSM and the likelihood of adverse pathology at the time of RP [57]. Decipher has also been incorporated into the development of a novel clinical-genomic risk stratification system that integrates GC results with traditional clinical parameters to redefine the National Comprehensive Cancer Network (NCCN) risk groups. This integrated model has been independently validated as a superior predictor of both metastasis and PCSM, significantly outperforming both the NCCN risk groups and the CAPRA score [58]. In addition to its established value for metastasis, PCSM, overall survival, and BCR risk, Decipher has evolved beyond its original role as a risk stratification tool to become a genomic biomarker with both prognostic and predictive utility for AS. It has been extensively validated for its ability to predict additional key clinical outcomes, including the presence of high-grade and high-stage disease at diagnosis, and the likelihood of disease progression following initial treatment, determining the appropriateness of AS versus definitive treatment in men with localized PCa [33,34,35,59]. In addition, recent evidence has linked Decipher scores with histopathologic upgrading, particularly in patients initially diagnosed with Gleason score 3+3 disease, supporting its role in refining patient selection for AS [60]. Furthermore, Decipher is emerging as a predictive biomarker for identifying patients who are likely to benefit from treatment intensification strategies or those responsive to specific therapeutic approaches [39]. Notably, in a recent analysis of the docetaxel arm of the STAMPEDE trial [40], Decipher showed significant predictive value: patients with high Decipher scores experienced a meaningful survival benefit when docetaxel was added to ADT, while those with low Decipher scores saw no benefit. Currently, Decipher Prostate is the most extensively validated genomic test in PCa, with support from over 80 peer-reviewed publications and clinical data from more than 200,000 patients. This robust body of evidence has led to its distinction as the only genomic test with level 1 evidence endorsed by the NCCN Guidelines for the management of localized PCa [27].

Compared to Prolaris and Oncotype DX GPS, a major advantage of Decipher is its use of transcriptome-wide RNA microarray technology, which enables the simultaneous assessment of over one million RNA expression signals. This broad profiling capability allows for the identification of more comprehensive molecular signatures, going well beyond the 22-gene panel used in other tests. This capability has led to the development of the Genomic Resource Information Database (GRID), a research-use-only resource that includes more than 200,000 whole-transcriptome profiles from patients with urologic malignancies [61]. This unique dataset enables large-scale exploration of gene expression patterns and supports the creation of novel molecular tools that can address unmet clinical needs in precision oncology. One notable example derived from the GRID platform is the Postoperative Radiation Therapy Outcomes Score (PORTOS), a predictive biomarker score specifically designed to identify patients who are most likely to benefit from postoperative RT [62]. While tools such as Decipher, CAPRA-S, and microarray-based cell-cycle progression signature did not predict differential benefit from RT, PORTOS emerged as the first predictive biomarker specifically capable of guiding RT dose personalization by identifying patients most likely to benefit from dose escalation, while helping to spare others from unnecessary treatment-related toxicity [62,63,64].

### 4.4. Scientific Gap

A key scientific gap of current GCs lies in their limited ability to account for spatial and molecular heterogeneity within the prostate. Recent spatial transcriptomic analyses have shown that tumors originating in the peripheral zone (PZ) and transitional zone (TZ) of the prostate exhibit distinct gene expression patterns and pathway activities. Notably, commercially available GCs yielded significantly different prognostic scores depending on the tumor zone. PZ tumors showed higher GC scores compared to TZ tumors, potentially reflecting a zone-dependent biological behavior rather than purely prognostic value [65]. In addition to intraprostatic heterogeneity, another key limitation is the geographic concentration of validation cohorts. Most GCs have been developed and validated in North American populations, with limited representation of other ethnic groups. Emerging validation studies in Asian cohorts have identified distinct gene expression profiles [66,67], raising concerns about the generalizability of GCs across diverse populations, particularly when based on bulk tissue data.

## 5. Current Guidelines Recommendations

Although definitive clinical utility through prospective randomized trials is unlikely due to feasibility constraints, the NCCN Guidelines v2.2025 [27] support the use of GCs in selected patients. Specifically, men with low- or favorable intermediate-risk PCa and a life expectancy of ≥10 years may consider the use of Decipher, Oncotype DX GPS, or Prolaris during initial diagnostic assessment. For patients with unfavorable intermediate- or high-risk disease and similar life expectancy, the use of Decipher or Prolaris is considered appropriate. In the post-RP setting, the Decipher assay is recommended as part of an individualized decision-making process when evaluating the need for adjuvant or salvage RT, particularly in the presence of adverse pathological features or PSA recurrence. It may also be considered for prognostication and risk stratification in cases of PSA persistence or BCR following surgery. Notably, as of NCCN Guidelines v2.2025, Decipher is currently the only gene expression test included under the “Advanced Tools” section for risk stratification and biomarkers [27].

Based on the recommendations discussed by the ASCO–EAU–AUA multidisciplinary expert panel [26] on the use of tissue-based biomarkers in PCa, the EAU/ESTRO 2025 [21] and the AUA/ASTRO 2022 [19] Guidelines state that these tests are promising, but have not yet been validated in prospective clinical trials, and that the impact of tissue heterogeneity and under sampling on prognostic accuracy remains a concern. However, the panel also acknowledges accumulating evidence indicating that GC scores (in particular Decipher) correlate with cancer outcomes [19]. Consequently, the guidelines emphasize that these tests should not be offered routinely, but rather used selectively in specific patient subsets where results may provide clinically actionable information, such as, for instance, in men with favorable intermediate-risk PCa considering AS, or those with unfavorable intermediate-risk PCa scheduled for RT, where treatment intensification with ADT may be considered.

Both the ASCO [26] and ESMO [20] guidelines acknowledge the potential value of tissue-based molecular assays in improving risk stratification and guiding treatment decisions in localized PCa, but do not recommend their routine use; instead, they support selective application when results are expected to influence clinical management, without endorsing specific GCs.

**Table 2:** Key validation studies.

GC	Setting and Tissue Type	Objective	Study
Prolaris	Post-treatment (RP tissue)	BCR and PCSM	Cuzick et al., Lancet Oncol 2011 [42]
		BCR	Cooperberg et al., JCO 2013 [41]
		Guidance for RT decision with/without ADT	Tward et al., Clin Genitourin Cancer 2021 [43]
		Response to dose-escalated RT	Tward et al., Int J Radiat Oncol Biol Phys 2022 [44]
		BCR and PCSM	Sommariva et al., Eur Urol 2016 [45]
	Pre-treatment (biopsy tissue)	AS eligibility	Cuzick et al., Br J Cancer 2012 [29]
		Long-term mortality	Cuzick et al., Br J Cancer 2015 [30]
		Support for the initial treatment decision	Hu et al., JCO PO 2018 [47]
		Metastatic risk	Hutten et al., JCO PO 2024 [31]
Oncotype DX GPS	Post-treatment (RP tissue)	Metastasis and PCSM	Brooks et al., JCO PO 2021 [51]
		Metastasis and PCSM	Van den Eeden et al., Eur Urol 2018 [52]
	Pre-treatment (biopsy tissue)	Adverse pathology	Cooperberg et al., J Urol 2013 [49]
		Adverse pathology	Klein et al., Eur Urol 2014 [33]
		AS eligibility	Badani et al., Urol Pract 2015 [32]
Decipher	Post-treatment (RP tissue)	Metastasis	Erho et al., PLoS ONE 2013 [36]
		Metastatic risk	Karnes et al., J Urol 2013 [38]
		Prognostic stratification	Ross et al., Eur Urol 2016 [59]
		BCR and metastasis post-RP RT	Den et al., Int J Radiat Oncol Biol Phys 2014 [55]
		Post-RP RT intensification benefit	Pollack et al., JCO 2025 [39]
	Pre-treatment (biopsy tissue)	Metastasis post-RP	Klein et al., Eur Urol 2015 [37]
		Metastasis post-RP	Klein et al., Urology 2016 [28]
		Metastatic risk post-RT+ADT	Nguyen et al., Prostate Cancer Prostatic Dis 2017 [56]
		AS selection	Zhu et al., Eur Urol Oncol 2024 [34]

GC = genomic classifier; PCSM = prostate cancer-specific mortality; BCR = biochemical recurrence; RP = radical prostatectomy; RT = radiotherapy; ADT = androgen deprivation therapy; AS = active surveillance.

## 6. Discussion

Although GCs have demonstrated clear clinical utility, as predictive and prognostic tools, their incorporation into standard care remains largely confined to scenarios where clinical ambiguity persists despite traditional risk stratification. In the context of low-risk disease, GCs may refine decisions regarding AS [29,30,33,34]. Similarly, in the post-RP setting, Decipher and Prolaris aim to provide decisional support where histopathology alone fails to stratify the need for adjuvant therapy [39,55,62]. More recently, Decipher and PORTOS scores have emerged as a potentially valuable instrument for evaluating treatment de-intensification strategies in advanced disease and guiding RT dose personalization, marking a shift from prognostic insight to true predictive utility [40,64]. The key point is that these tools have the potential not merely to improve risk classification, but to actively minimize overtreatment in patients with molecularly indolent tumors while guiding more appropriate treatment intensification in those with aggressive disease. The implication is that GCs should be prioritized not where risk is obvious, but precisely where clinical equipoise exists. The paradox, however, is that despite being championed as hallmarks of precision oncology, their real-world adoption remains uneven, driven less by evidence-based thresholds than by institutional heterogeneity, access disparities, and economic constraints. In contrast to GCs, PRSs, whose deployment is still primarily research-driven, target the pre-diagnostic population, positioning themselves as a genomic layer atop conventional screening tools. While not yet integrated into therapeutic pathways, offer a promising approach for identifying men at elevated inherited risk, but despite substantial advances, they are also subject to important constraints. Their predictive performance varies significantly across ancestral populations, with reduced accuracy in non-European groups, raising concerns about equity and potential exacerbation of health disparities. Furthermore, PRSs do not capture rare, high-penetrance mutations, thereby omitting a significant portion of inherited cancer risk. In PCa specifically, PRSs have not yet demonstrated sufficient ability to distinguish between indolent and aggressive disease. For example, in the BARCODE1 trial [17], while many cancers identified through PRS-based screening were intermediate or high risk, a substantial proportion were still classified as low-risk, raising concerns about possible overdiagnosis [68]. One of the main limitations of genomic tools remains their high upfront cost, which poses challenges to widespread adoption, particularly in cost-sensitive health systems. Nonetheless, several economic evaluations have shown that genomic testing can be cost-effective in selected clinical scenarios, particularly when it leads to reduced overtreatment.

For example, PRS use has been shown to significantly reduce overdiagnosis compared to age-based approaches, while offering a more favorable cost-benefit profile. Specifically, risk-based PSA screening at a 10-year absolute risk threshold of ≥4% was found to be more cost-effective and associated with fewer overdiagnoses, with only a modest reduction in prostate cancer deaths averted [69]. Regarding GC, a cost-utility analysis of Decipher in the post-RP setting showed that GC-guided decisions on adjuvant RT reduced the risk of distant metastases by 16% over five years and were cost-effective and were considered cost-effective when the test was priced at $4000, remaining cost-saving up to a test price of $11,402 [70]. Similarly, a prospective study evaluating Oncotype DX GPS found that its use led to a higher rate of AS (59% vs. 38%) and reduced use of RT, resulting in overall cost savings and improved efficiency of care [53]. An interesting decision-analytic model evaluated the economic impact of implementing the Prolaris test in a US commercial health plan for men with localized PCa eligible for AS [71]. The analysis showed that introducing the test into a plan covering five million members would result in approximately $4.5 million in savings over three years. These savings were primarily attributable to increased uptake of active surveillance and decreased use of unnecessary definitive treatments, underscoring the potential of genomic tools to support more cost-effective, risk-adapted management in low-risk prostate cancer. While these findings are promising, a key aspect must be acknowledged: all available cost-effectiveness data originate from the US healthcare system, which is characterized by high treatment costs and market-driven pricing. This limits the direct applicability of such results to classic European public healthcare and elsewhere. Region-specific economic evaluations are needed to assess whether similar cost-benefit profiles can be achieved in publicly funded contexts, where cost structures, clinical pathways, and reimbursement dynamics differ substantially.

Behavioral responses to genomic tools disclosure also remain poorly understood. For instance, receiving a low-risk PRS result may lead to reduced adherence to recommended screening or delayed medical consultation, with uncertain long-term consequences [72]. Besides the established tools, a growing number of emerging molecular assays, such as urine-based tests [73,74], exosomal biomarkers [75,76], and platforms like the Prostatype P-score [77,78,79], have shown promise for early detection and refined risk stratification. However, these remain outside current clinical guidelines due to limited validation and the absence of robust prospective evidence. At the same time, future developments in omics technologies and AI-assisted biomarker discovery are expected to significantly enhance the precision of genomic tools. While these innovations reflect the rapid pace of advancement in precision oncology, their incorporation into routine care should await stronger clinical validation. As precision oncology continues to advance, both GCs and PRSs are expected to gain wide integration into clinical practice. Specifically, GCs are likely to evolve beyond prognostic tools to become key predictors of treatment response, as the PORTOS score is already doing [62,64], and a key factor in treatment selection. Platforms such as Decipher GRID [61] offer an incredible opportunity for this path by enabling genomic data analysis for novel biomarker discovery and personalized therapeutic targeting.

In parallel, large-scale initiatives such as the UK’s Our Future Health program [80], which aims to recruit up to five million participants and return genomic risk information, are expected to play a pivotal role in refining PRS models and facilitating their clinical integration. Ultimately, increased standardization, improved cost-effectiveness, and growing familiarity among clinicians may help overcome current barriers and position genomic testing as a cornerstone of personalized care in prostate cancer.

## 7. Conclusions

While GCs are actionable tools for patients with established PCa, providing robust prognostic and predictive information that can inform treatment decisions, PRSs offer a promising approach for identifying men at elevated PCa inherited risk. Both tools add value where conventional clinical parameters reach their limits, enhancing prognostic precision, individualized care, and decision-making. Nevertheless, widespread implementation remains limited by economic considerations, and further validations through prospective cohort studies are needed to establish their clinical relevance. If GC use is justified where clinical uncertainty exists, PRS application is most compelling where screening ambiguity dominates. When applied selectively and appropriately, these tools represent a key advancement toward truly personalized and precision-based PCa care.

## Figures and Tables

**Table 1:** Main characteristics of Genomic Classifier tests.

GC	Prolaris	Oncotype DX GPS	Decipher
Commercial Provider	Myriad Genetics	MDxHealth	Veracyte
Technology	RT-PCR	RT-PCR	Whole-transcriptome RNA microarray
Number of Genes	31 CCP genes + 15 housekeeping	17 genes (12 cancer-related + 5 reference)	22 genes panel + transcriptome-wide data
Tissue Source	FFPE tissue from biopsy or RP	FFPE tissue from biopsy or RP	FFPE tissue from biopsy or RP
Score Output	1.8–8.7	0–100	0–1
Setting	Pre- and post-treatment	Pre-treatment	Pre- and post-treatment; RT, RP, ADT guidance
NCCN Guidelines 2025	Selective use in low/intermediate risk	Selective use in very low/low/intermediate risk	Supported for all risk groups; part of “Advanced Tools”. level 1 evidence

GC = genomic classifier; RT = radiotherapy; RT-PCR = reverse transcription polymerase chain reaction; RNA = ribonucleic acid; CCP = cell-cycle progression; FFPE = formalin-fixed paraffin-embedded; RP = radical prostatectomy; ADT = androgen deprivation therapy; NCCN = National Comprehensive Cancer Network.
